# False Positive FDG PET/CT Resulting from Fibrous Dysplasia of the Bone in the Work-Up of a Patient with Bladder Cancer: Case Report and Review of the Literature

**DOI:** 10.5812/iranjradiol.10303

**Published:** 2012-12-27

**Authors:** Mustafa Aras, Tunc Ones, Faysal Dane, Omid Nosheri, Sabahat Inanir, Tanju Yusuf Erdil, Halil Turgut Turoglu

**Affiliations:** 1 Department of Nuclear Medicine, Marmara University School of Medicine, Istanbul, Turkey; 2 Department of Internal Medicine, Oncology Division, Marmara University School of Medicine, Istanbul, Turkey

**Keywords:** Fibrous Dysplasia, Polyostotic, Bone and Bones, 18F-Flourodeoxyglucose, PET, CT

## Abstract

Fibrous dysplasia of the bone (FDB) is a common, genetic, developmental disorder with a benign course. FDB can be seen anywhere throughout the skeleton. It is usually asymptomatic and found incidentally on imaging studies that are performed for other purposes. Although whole body 18 F-flourodeoxyglucose PET/CT (FDG PET/CT) is widely used in tumor imaging, infections and benign pathologies like FDB may cause false positive results. Herein we report the case of a 48-year-old FDB patient with transitional cell carcinoma of the urinary bladder. Restaging FDG PET/CT showed multiple mild to moderate hypermetabolic bone lesions which were initially misinterpreted as bone metastases. In this case report, we aimed to guide physicians in evaluating bone lesions in cancer patients with FDB in the light of the literature.

## 1. Introduction

Fibrous dysplasia of the bone (FDB) is a slowly progressive bone disorder in which normal bone is replaced by abnormal fibro-osseous tissue (1, 2). The true incidence and prevalence rates of FDB are difficult to estimate, but the lesions are not rare (3). The disease may involve single bone (monostotic – 60%) or multiple bones (polyostotic – 40%) with a predilection for the craniofacial bones, ribs, pelvis and long bones (2, 3).

FDB is usually asymptomatic and found incidentally on imaging studies that are performed for other purposes (3). Malignant transformation may also be seen in some FDB patients and the risk is increased by radiotherapy (3).

Whole body 18 F-flourodeoxyglucose PET/CT (FDG PET/CT) has been widely used in tumor imaging recently. Several FDG PET/CT reports suggested that the appearance of FDB could mimic a malignant process. Therefore, this case was presented with the aim of guiding physicians in evaluating bone lesions in cancer patients with FDB.

## 2. Case Presentation

**Figure 1:**
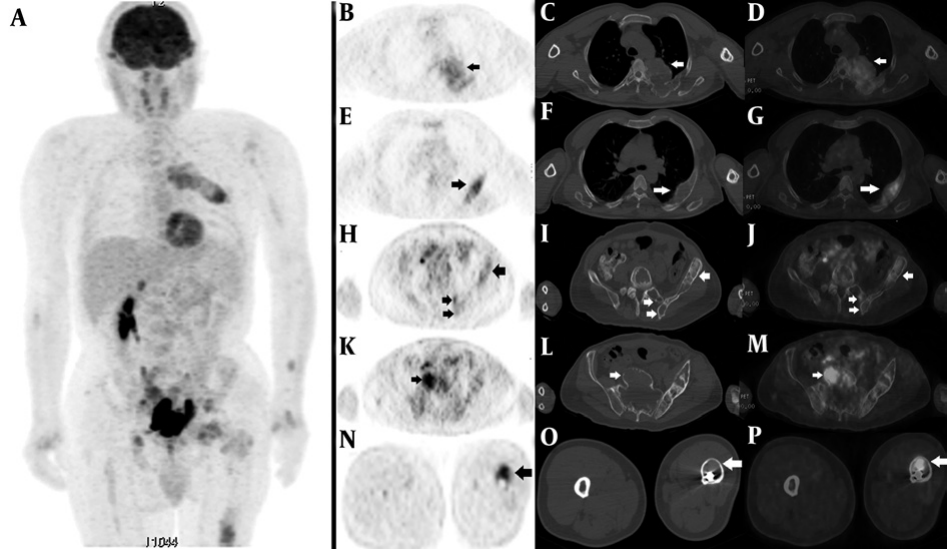
A, Maximum intensity projection (MIP) image; B, E, H, K, N: axial FDG PET images; C, F, I, L, O: axial CT images; D, G, J, M, P: axial fused FDG PET/CT images. FDG PET/CT images showed lytic, expansile, mild to moderate heterogeneous hypermetabolic bone lesions in the left hemithorax, left hemipelvis and left lower extremity.

**Figure 2:**
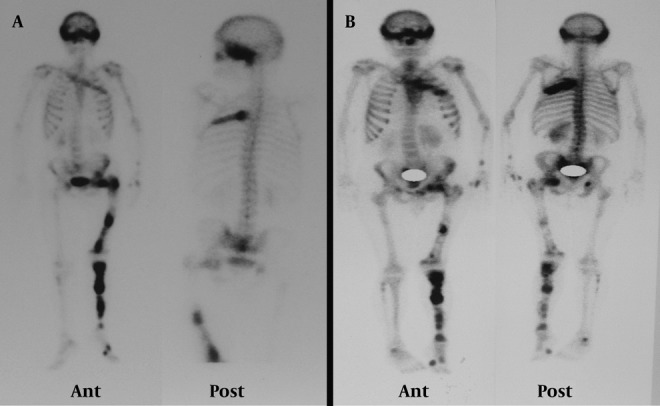
Patient’s previous whole body bone scan (A) showed increased osteoblastic activity in the cranium, left hemithorax, bilateral upper and lower extremities and pelvic bones. The recent bone scan (B) shows that the polyostotic FDB lesions are compatible with the previous bone scan and the recent FDG PET/CT scan.

A 48-year-old man presented with gross hematuria to the urology service. Abdominopelvic ultrasonography revealed a tumoral mass lesion in the left inferolateral wall of the urinary bladder. Transurethral resection of the mass was performed and the pathology was reported as transitional cell carcinoma. FDG PET/CT was requested for restaging. It showed lytic, expansile, moderate to intense hypermetabolic bone lesions [maximum standardized uptake value (SUVmax):5.5] in the cranium, left hemithorax, left hemipelvis and left lower extremity (Figure 1). When the past medical history of the patient was reviewed in detail, it revealed polyostotic FDB diagnosed 10 years ago. The blood test showed an increase of the alkaline phosphatase to 114 U/l (reference value: 35-104 U/l), otherwise with parameters of normal value. He did not describe any other symptoms suggestive for FDB. We reviewed the patient’s previous bone scintigraphy which was performed 10 years ago (Figure 2A). It showed increased osteoblastic activity in the cranium, left hemithorax, bilateral upper and lower extremities and pelvic bones. Later, a new bone scan was requested to reevaluate the polyostotic FDB lesions (Figure 2B). When these three imaging studies were reviewed together, the PET/CT appearances of the lesions had similarities to those on the scintigraphic ones. These overlapping findings were interpreted in favor of FDB. Furthermore, the costal lesion in the left hemithorax (which persisted in all three imaging studies) was biopsied and reported as FDB. The verified FDB lesions in this patient prevented him from receiving unnecessary chemotherapy.

## 3. Discussion

Conventional imaging methods (X-ray, CT and MRI) have been used to diagnose FDB. The imaging features of FDB are characteristic, although not specific and these lesions without periosteal reaction or soft tissue involvement generally look benign (2, 3). Additionally, FDG PET/CT; Tc 99 mMDP; Ga 67 Citrate, In 111 pentreotide, In 111 leukocyte scintigraphy; and dual phase Tc 99 mMIBI scintigraphy imaging have also been used for the evaluation of FDB (4). New bone formation and increased vascularity suggest an active osteoblastic state within the lesions of FDB, resulting in increased uptake of bone imaging agents (5).

Several case reports have been described that FDB can have either intense FDG activity or can be metabolically normal without any increased FDG activity on PET-CT scan. Variability of FDG-uptake in various sites in one lesion or an individual may be due to differing numbers of actively proliferating fibroblasts (6). The significantly increased FDG uptake may mimic bone metastasis or skeletal involvement of the primary malignancy in cancer patients with FDB. Most of the studies in the literature dealing with cancer patients with FDB are presented in Table 1. These findings may change the stage of the cancer as in our case. Toba et al. concluded that the growth of FDB lesions needed the acceleration of bone mineral turnover without an increase in glucose metabolism (7). Charest et al. reported a patient with synchronous liposarcoma and a monostotic FDB lesion; the SUV max of the monostotic lesion (proved by biopsy) was higher (8). Su et al. reported that early SUV max values in the lesions of FDB ranged from 1.2 to 9.6 for 11 patients and this variability may cause higher SUV max values for FDB lesions compared to malignant lesions as in Charest report (6, 8).

**Table 1:** Review of the Literature in Cancer Patients with FDB

Authors	Type of Article	Year	Subject
Shigesawa et al.	Case Report	2005	FDG PET/CT is useful for detecting and differentiating bone metastasis from FDB
Berrebi et al.	Case Report	2008	FDG PET/CT is useful in the early diagnosis of malignant transformation of FDB
Su et al.	Research Article	2010	Higher SUVmax values reported for the FDB lesions than the malignant lesions
Kim et al.	Case Report	2009	False (+) FDG PET/CT findings
Charest et al.	Case Report	2008	
Bonekamp et al.	Case Report	2008	
Ho et al.	Case Report	2006	
Stegger et al.	Case Report	2007	
Kao et al.	Case Report	2007	
von Falck et al.	Case Report	2008	
Strobel et al.	Case Report	2007	
Basu et al.	Case Report	2010	

On the other hand, Shigesawa et al. showed that FDG PET/CT is useful for differentiating bone metastasis from FDB in patients with a malignancy (9). Whole body bone scintigraphy remains the best method to identify the extent of skeletal involvement especially in polyostotic FDB. Zhibin et al. (10) reported that the diagnostic specificity of ruling out metastases with radionuclide bone scanning may be improved in association with other imaging modalities (10).

Malignant transformation including osteosarcoma, fibrosarcoma and chondrosarcoma may also be seen in some FDB patients and the risk increases by radiotherapy (3). Berrebi et al. (11) concluded that the local progressive increase of the SUV max index can contribute to an early detection of sarcomatous transformation. However, Bonekamp et al. (12) described a case of biopsy-proven fibrous dysplasia of the skull in a colon cancer patient which changed its FDG activity and CT appearance within 10 months of follow-up (12).

CT is the best technique for demonstrating the radiographic characteristics of FDB (3). The most common appearance of FDB on CT is an expanded bone showing a ground-glass appearance (3, 13, 14). Strobel et al. (15) reported that dedicated CT interpretation led to the correct diagnosis of a benign lesion (15). However, FDB may mimic other benign fibro-osseous lesions and may even be confused with certain types of malignancies (3, 13, 16-18). The CT appearance of FDB is in proportion with the extent of mineralization (18). Lesions with increased CT density may be undergoing mineralization; while in lower CT density lesions, fibroblastic proliferation may predominate (19). Su et al. (6) reported that the peak SUV max values in FDB lesions were often located in the area with the lowest density lesions. Other diffusely ossifying or calcifying lesions may have a similar appearance on CT scans (18). FDB usually has sclerotic margins on CT scans and may have a matrix of uniform density (17). Additionally, CT can reveal areas of cortical breakthrough usually associated with cortical thinning and bone expansion that are not appreciated occasionally on radiographic reviews (17, 20-23). Distinction between FDB, adamantinoma and ossifying fibroma can be difficult and FDB can appear very similar to adamantinoma (13, 14, 16, 24-26). This distinction can be made pathologically (13). Other radiologic differential diagnoses include simple bone cyst, nonossifying fibroma, paget disease, low-grade intramedullary osteosarcoma, giant cell tumor, neurofibromatosis and osteoblastoma (3, 13, 18).

In the light of these literature findings, it might be concluded that FDG PET/CT scans need to be interpreted in the overall clinical context, on a patient-per-patient basis. Cho et al. (27) concluded that MRI in addition to radiography may help differentiate fibrous dysplasia from metastasis in patients with malignancy. In our case, new bone scintigraphy was performed instead of conventional imaging methods to confirm the diagnosis. The recent bone scan showed the lesions that correlated with the ones on both the previous bone scan and FDG PET/CT scan. The CT characteristics of the lesions on FDG PET/CT were also compatible with FDB.

In conclusion, if the FDG positive bone lesions are the only positive findings on PET/CT scan, except for the primary tumor in cancer patients with a past medical history of FDB; they should not be misinterpreted as bone metastases. To improve the diagnostic accuracy, such lesions need to be correlated with the previous imaging studies and biopsied if they are equivocal.
